# Three-Dimensional Metal-Oxide Nanohelix Arrays Fabricated by Oblique Angle Deposition: Fabrication, Properties, and Applications

**DOI:** 10.1186/s11671-015-1057-2

**Published:** 2015-09-21

**Authors:** Hyunah Kwon, Seung Hee Lee, Jong Kyu Kim

**Affiliations:** Department of Materials Science and Engineering, Pohang University of Science and Technology (POSTECH), Pohang, 790-784 South Korea

**Keywords:** Three-dimensional nanostructured thin films, Oblique angle deposition

## Abstract

Three-dimensional (3D) nanostructured thin films have attracted great attention due to their novel physical, optical, and chemical properties, providing tremendous possibilities for future multifunctional systems and for exploring new physical phenomena. Among various techniques to fabricate 3D nanostructures, oblique angle deposition (OAD) is a very promising method for producing arrays of a variety of 3D nanostructures with excellent controllability, reproducibility, low cost, and compatibility with modern micro-electronic processes. This article presents a comprehensive overview of the principle of OAD, and unique structural and optical properties of OAD-fabricated thin films including excellent crystallinity, accurate tunability of refractive indices, and strong light scattering effect which can be utilized to remarkably enhance performances of various systems such as antireflection coatings, optical filters, photoelectrodes for solar-energy-harvesting cells, and sensing layers for various sensors.

## Review

### Introduction

Over the last few decades, various nanostructures have received steadily growing interests for many applications due to their fascinating physical, optical, and chemical properties [1–4]. Novel physical properties such as size-dependent excitation or emission, quantized conductance, and metal-insulator transition have been reported to emerge when the size of structures is reduced to nanoscale dimensions [5–11]. In the field of photonics, natural or engineered nanostructures often cause novel optical phenomena based on the interaction between light and nanostructured materials with specific geometrical shape, size, orientation, and arrangement [12]. In addition, the highly porous nature of nanostructured films having giant surface areas for chemical reactions, together with nanoscale dimensions comparable to the Debye length, can bring about innovation in various fields such as chemical sensor systems [13–17] and photoelectrodes for solar-energy-harvesting cell systems [18–24]. Besides, properties of nanostructures can be diversely tailored by controlling their sizes and structures providing tremendous opportunities to be applied in a wide variety of research fields [25–27].

Nanostructures can be classified according to the dimensionality of the nanoscale component: zero-dimensional (0D, nanoparticles, quantum dots, etc.) [9–11], one-dimensional (1D, nanowires, nanorods, etc.) [8, 13], two-dimensional (2D, nanoplates, nanoscale multilayers, etc.) [7, 28], and three-dimensional (3D, nanohelixes, various hierarchical structures) [29, 30] nanostructures. Among them, 3D nanostructures can have larger degree of freedom in design than other nanostructures by precisely controlled structural factors such as geometrical shape and size, opening possibilities for realization of multifunctionality and ultimate performance of various systems, and exploring new physical phenomena [29, 31, 32]. Therefore, fabrication of thin films based on 3D nanostructures with a precisely controlled and reproducible way is of greatest interest to many researchers. Several fabrication methods for various 3D nanostructures have been developed such as colloidal self-assembly [33], holographic laser lithography [34], phase-mask holography [35], layer-by-layer fabrication [36, 37], and direct writing techniques [38, 39]; however, they are somewhat complex, costly, and lacking in compatibility with conventional microelectronics fabrication processes for introducing such nanostructures into integrated circuit chips.

Oblique angle deposition (OAD), or often referred to as glancing angle deposition (GLAD), is a method for producing an array of 3D nanostructures with excellent controllability in geometrical shape, reproducibility, low cost, and compatibility with current microelectronics fabrication techniques [40–46]. By controlling deposition parameters during OAD, a variety of nanostructures can be fabricated including slanted nanorods and nanohelixes (NHs). In particular, arrays of metal-oxide NHs are promising building blocks due to their unique structural and optical properties which can be adjusted precisely to satisfy specific requirements for various future applications. In this article, recent studies on 3D nanostructures of various metal oxides fabricated by OAD are reviewed. Firstly, the fabrication principle of various nanostructures by OAD is introduced. Secondly, unique structural and optical properties of 3D NH arrays are discussed including crystallinity, tunability of refractive index, and light scattering effects. Finally, promising applications of nanostructures fabricated by OAD such as passive optical components, photoelectrodes for solar-energy-harvesting cell systems, and sensing layers for chemical sensors are reviewed.

### Fabrication of 3D Nanostructured Thin Films by OAD

**Fig. 1:**
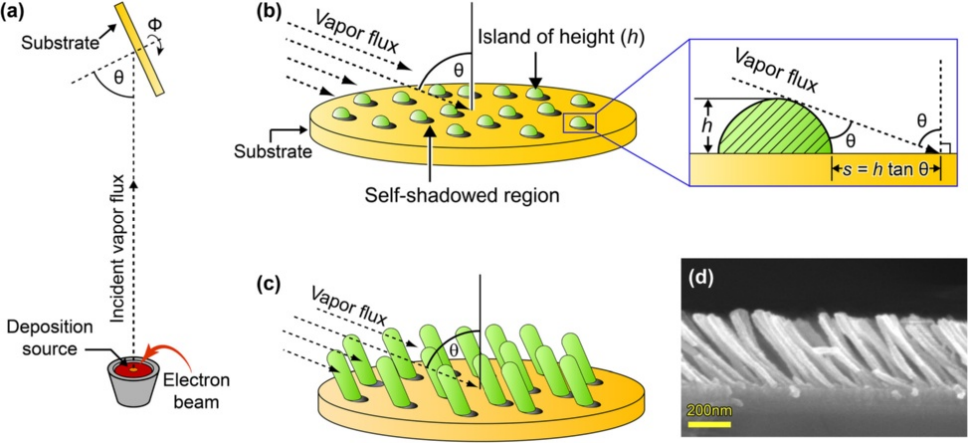
Oblique angle deposition. **a** Schematic representation of oblique angle deposition (OAD) by e-beam evaporation. The angle between the substrate normal and vapor flux is *θ*, and the substrate rotation along the substrate normal axis is *ϕ*. **b** Islands of depositing materials (with the height of *h*) are formed on the substrate at the initial stage of OAD. The magnified view shows self-shadowed regions (*h* tan *θ*) formed by highly oblique angle of vapor flux. **c** Highly porous nanorod arrays fabricated by OAD. **d** Cross-sectional SEM image of SiO_2_ nanorod film with a deposition angle of 80°

OAD, a geometrical deposition process which uses a highly directional vapor flux source to create self-organized nanoporous thin films, is a low-cost and highly versatile nanofabrication technique to tailor a wide variety of nanostructures of a wide range of available materials such as metals, semiconductors, and insulators [40–46]. In this method, vapor flux has an incident angle (*θ*) with respect to the substrate surface-normal direction by tilting the substrate, as schematically shown in Fig. 1a. At the initial stage of the deposition, incident vapor flux forms nanoscale nuclei randomly distributed on the substrate, and the initial nucleated islands provide a self-shadowed region where subsequent incident vapor flux can no longer deposit, as shown in Fig. 1b. As can be seen in the inset of Fig. 1b, the shadowed length is given by *h* tan *θ* for a nucleus with height *h* [44]. As the deposition proceeds, limited atomic diffusion into the self-shadowed region and preferential deposition of the vapor flux on top of the islands create porous slanted nanorod arrays shown in Fig. 1c. Figure 1d shows a cross-sectional scanning electron microscopy (SEM) image of a uniform array of SiO_2_ nanorods deposited on Si substrate with a deposition angle of 80° without rotation.

**Fig. 2:**
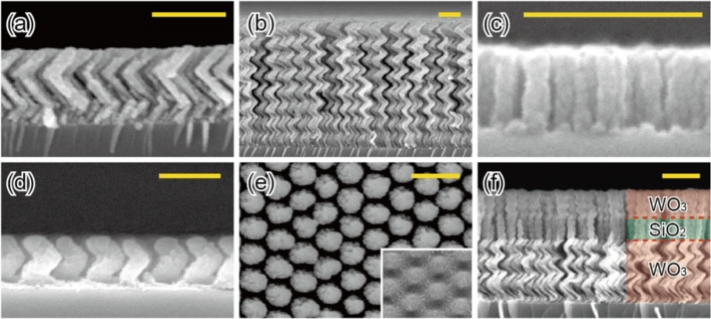
Various nanostructured thin films by OAD. Cross-sectional and top-view SEM images of various nanostructured films fabricated by OAD. *Scale bar* = 500 nm. **a** SnO_2_ zigzags, **b** TiO_2_ helixes, **c** Al vertical posts, and **d** WO_3_ helixes grown on ZnO pre-patterns by nanoimprint lithography. **e** Top-view SEM image of WO_3_ helixes grown on pre-patterned substrate. *Inset* shows top-view SEM image of pre-patterned ZnO by nanoimprint lithography. **f** WO_3_-SiO_2_-WO_3_ hetero-NHs

The geometrical shape of 3D nanostructures can be precisely controlled by adjusting deposition conditions such as the vapor flux incidence angle (*θ*), the substrate rotation speed (*ϕ*), and the deposition rate (*r*) during OAD [47]. Figure 2a–c shows cross-sectional SEM images of SnO_2_ nano-zigzags, TiO_2_ NHs, and Al vertical posts, respectively, the geometrical shapes of which are tailored by adjusting *r*, *θ*, and *ϕ*. Especially, 3D NHs can be formed when the substrate rotation speed is intentionally set to provide the vapor flux with sufficient time to deposit along all the direction changing with time. Either a high *r* or a fast *ϕ*, or a combination of both can result in the formation of vertical posts rather than NHs, as shown in Fig. 2c.

In addition to the three parameters, *r*, *θ*, and *ϕ*, determining the geometrical shape of the nanostructures, the temperature, and the surface morphology of substrates is also important as they influence the porosity and the periodicity of nanostructured thin films. Since substrate temperature affects the diffusion of depositing vapor flux on the substrate at the initial stage as well as on top of the nuclei, it is strongly related to the density of nanostructured thin films and the dimension of individual nanostructures constituting the thin film. Furthermore, introducing periodically ordered topographical patterns on the substrate can produce the periodically ordered nanostructures enabled by the formation of “enforced” nucleation sites. Figure 2d,e shows cross-sectional and top-view SEM images of Si NHs on a ZnO pre-patterned substrate, respectively. The inset of Fig. 2e shows a top view of the ZnO pre-pattern on Si substrate achieved by nanoimprint lithography [48]. The Si NHs exhibit a periodic hexagonal close-packed array due to the periodicity of ZnO pre-patterns acting as the initial nucleation seeds. Enforcing periodically arranged nucleation sites for OAD can be a very elegant way to realize a novel optical phenomenon caused by a periodicity of 3D nanostructures [49–56].

One of powerful aspects of OAD is its ability to fabricate heterostructures consisting of various materials such as oxides, metals, and insulators with various combinations of nanostructures. Although a great deal of efforts has been made to fabricate aligned heterostructures in nanoscale by using conventional nanofabrication methods, the formation of 3D nano-heterostructures is quite difficult due to limitations in the method itself as well as available materials suitable for the method. Figure 2f shows the NH heterostructure consisting of WO_3_-SiO_2_-WO_3_ with different geometrical shapes. NHs of a material can be sequentially grown on NHs of another material which acts as the nucleation sites. Note that 3D nano-heterostructures based on various combinations of materials, for example, metal-oxide, oxide-oxide, semiconductor-oxide, etc., can be fabricated on demand, which can exhibit new functionalities with tailored electric, magnetic, optical, and mechanical properties [57–59].

### Properties of 3D NH Arrays

#### Structural Properties

**Fig. 3:**
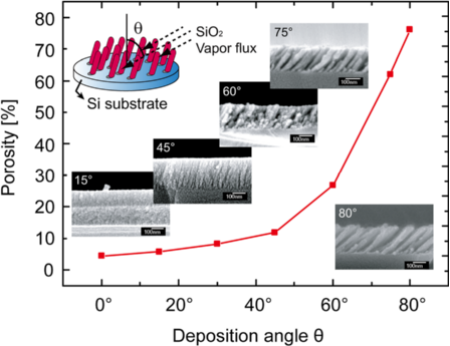
Porosity of SiO_2_ thin films as a function of the vapor incident angle, estimated from measured refractive indices of thin films. *Inset* images show cross-sectional SEM images of films

Nanostructured thin films fabricated by OAD technique are typically porous. Besides substrate temperature, the vapor flux incident angle *θ* predominantly affects the density of nuclei at the initial stage of OAD. As *θ* increases, the density of nuclei decreases, resulting in the formation of a thin film with high porosity. An analytic model that accurately predicts the porosity and deposition rate of nanostructured thin films was proposed by Poxson et al. [44]. The porosity of a thin film can be estimated from measured refractive index of the thin film using the Bruggemann effective medium approximation [60, 61] which gives effective refractive index of the thin film consisting of two components, air and the depositing material. Figure 3 shows the calculated porosity of SiO_2_ thin films fabricated by OAD as a function of *θ*, clearly showing that porosity increases as *θ* increases. As shown in the SEM images in Fig. 3, the SiO_2_ thin films become more porous with increasing *θ*. High porosity and surface-to-volume ratio of nanostructured thin films fabricated by OAD can provide a great potential to be used for active layers of highly sensitive chemical sensors where the area of the chemically active surface is a critical factor determining the performance.

**Fig. 4:**
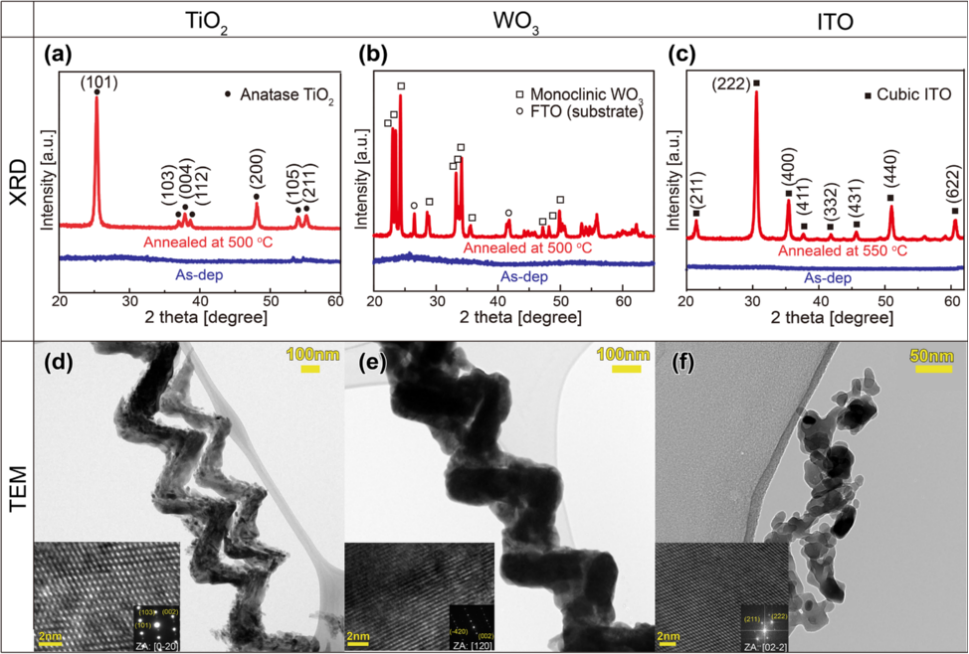
Micro-structural properties [21–24]. **a–c** XRD patterns and **d–f** TEM images (*inset*: high-resolution TEM images and diffraction patterns) of TiO_2_, WO_3_, and ITO NHs. All as-deposited NHs are amorphous films, while they become highly crystalline after annealing (TiO_2_ NHs: 500 °C for 30 min, WO_3_ NHs: 500 °C for 1 h, ITO NHs: 550 °C for 1 min)

The dimension of an individual nanostructure constituting the porous thin film is mainly determined by the material properties including the diffusion coefficient and substrate temperature. The typical dimension of the individual nanostructure fabricated by OAD at room temperature is ranged from tens of nanometers to ~200 nm, as characterized by SEM images in Fig. 2, depending on the surface diffusivity of the depositing material. The crystallographic phase of nanostructures can be characterized by X-ray diffraction (XRD) and transmission electron microscopy (TEM). Figure 4a–c shows XRD patterns measured from arrays of TiO_2_, WO_3_, and ITO NHs. No diffraction peak is observed for the as-deposited NHs, indicating that the as-deposited NHs by OAD using electron-beam evaporation are in the amorphous state. After annealing the NH arrays (TiO_2_ NHs: 500 °C for 30 min, WO_3_ NHs: 500 °C for 1 h, ITO NHs: 550 °C for 1 min), diffraction peaks corresponding to crystallographic planes of the anatase TiO_2_, monoclinic WO_3_, and cubic ITO, respectively, appear, indicating that thermal annealing of NHs induces phase transformation from the amorphous to crystalline phases. The preferred orientations of the anatase TiO_2_ NHs, quantitatively evaluated from the XRD pattern by calculating from peak intensity, are (103) and (101), which is consistent with TEM results [21]. Figure 4d–f shows TEM images of individual TiO_2_, WO_3_, and ITO NHs with their high-resolution TEM images and corresponding electron diffraction patterns in the insets. The TiO_2_ NH and the WO_3_ NH are found to be composed of a large near-single-crystalline domain with a few grain boundaries throughout the whole NH, whose average grain size corresponds to ~1.5 times the pitch of the NH along the growth direction [21, 22]. On the other hand, ITO NHs are composed of randomly oriented small, but highly crystalline grains.

A large near-single-crystalline domain with a few grain boundaries over the individual NH can ensure an excellent electrical conduction with low carrier recombination losses at grain boundaries, which can be utilized for electrodes of various devices where charge collection efficiency needs to be enhanced. Furthermore, high porosity in NH arrays allows the possibility of fabricating hybrid structures, for example, by conformal coating of individual NHs with other materials or by filling 0D or 1D nanomaterials into the pores between NHs. Such hybrid structures composed of NH arrays and other materials have a great potential to be applied for a variety of high-performance devices due to their multifunctionality and excellent design freedom. Examples of applications of NH arrays or their hybrid structures as electrodes will be introduced in the section “Applications of 3D NH arrays”.

#### Optical Properties

**Fig. 5:**
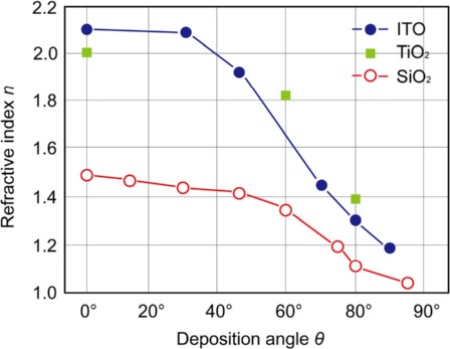
Tunability of refractive indices. Refractive indices of ITO, TiO_2_, and SiO_2_ thin films as a function of the vapor incident angle. ITO at *λ* = 474 nm, TiO_2_ at *λ* = 550 nm, and SiO_2_ at *λ* = 460 nm

In fields of optics and photonics, the refractive index of a medium is the most fundamental quantity, thus a key material property determining performance in a variety of applications such as antireflection coatings, Bragg reflectors, photovoltaic cells, and light emitters [58, 61–66]. Thin film materials containing a high density of nanoscale pores can be considered as a composite of the depositing material and air; therefore, their refractive indices are different from those of the dense materials. Since the porosity of thin films fabricated by OAD is varied according to *θ* as shown in Fig. 3, the refractive indices can be also precisely controlled. Figure 5 shows the measured refractive indices of thin films composed of TiO_2_, ITO, and SiO_2_ slanted nanorods fabricated by OAD with varying *θ*. At low *θ*, the self-shadowed region cannot be formed, resulting in the deposition of dense thin film with refractive index of the dense material. With increasing *θ*, the porosity increases due to the expanded self-shadowed region; therefore, the refractive index of the thin film decreases. The refractive indices of thin films shown in Fig. 5 vary from their dense phase values down to unprecedented low values of 1.05 [61]. Note that dense materials with very low refractive indices (*n* < 1.39) do not exist although modern integrated optical structures require access to materials covering a wide range of refractive indices. MgF_2_ is a material with the refractive index among the lowest available but its refractive index is 1.39, much higher than that of air. The unique ability of control over the refractive index of thin film materials allows one to *close the refractive index gap* between 1 (*n*_air_ ~ 1) and ~1.39 (*n*_MgF2_) at visible wavelengths, thus to overcome *fundamental* limitations in the realization of ultimate performance of optical components and optoelectronic devices due to the unavailability of materials with desired refractive indices [61]. Examples of this discussion will be presented in the “Applications of 3D NH arrays”.

**Fig. 6:**
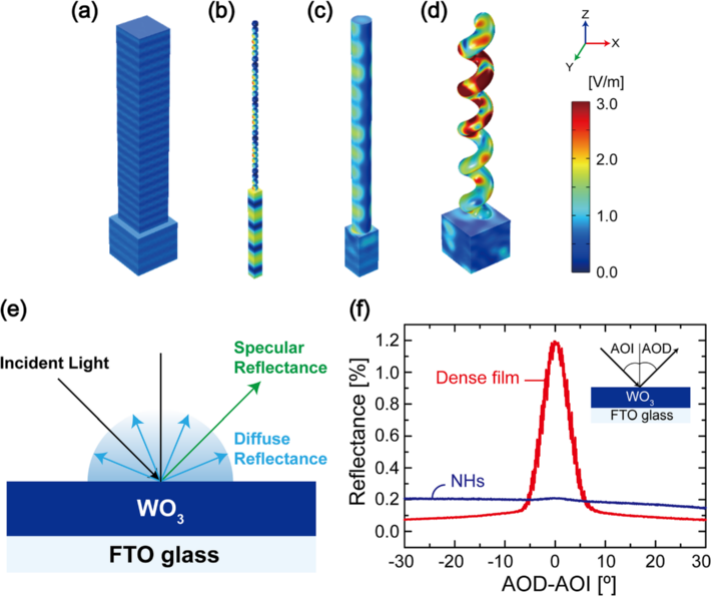
Light scattering effects [24]. **a–d** Distribution of the electric field magnitude at the WO_3_ surfaces of **a** a thin film (2.4-μm height), **b** a stack of nanoparticles (30-nm diameter, 30 nanoparticles), **c** a nanorod (100-nm rod diameter, 2.4-μm height), and **d** a NH (100-nm rod diameter, 400-nm helix diameter, 2.4-μm height) on a 500-nm FTO film. The *scale bar* on the right represents the magnitude of the electric field. **e** A schematic of specular and diffuse reflectance and **f** measured specular and diffuse reflectance of a 6-μm WO_3_ dense film and of the WO_3_ NH array as a function of the difference between the angle of the detector (AOD) and the angle of incidence (AOI) (30°). The *inset* shows the schematic measurement configuration

Among various nanostructures available by OAD method, 3D NH arrays have a great advantage in terms of light scattering. 3D NH arrays can increase interface areas between the curled nanostructures and air where the incident light can be scattered—in other words, the light scattering cross section for the NHs is much larger than that for other nanostructures [21, 22]. In order to investigate the effect of the 3D NH array on light scattering, Shi and Choi et al. [24] performed finite elemental simulations (Fig. 6a–d). The magnitude of the electric field on the surface of WO_3_ 3D NHs (Fig. 6d) is ~3-fold larger than that of thin film, stacked nanoparticles, and 1D nanorod (Fig. 6a–c, respectively). In addition, more complex distribution of the electric field is founded on the NHs, indicating much enhanced light scattering by the 3D NH arrays. The degree of light scattering by the 3D NH arrays was experimentally compared with that of dense thin film by specular and diffuse reflectance measurements. Figure 6e is a schematic diagram showing specular and diffuse reflecting rays when incident light impinges on WO_3_ thin films—dense WO_3_ and an array of WO_3_ NHs on a glass substrate. Specular reflectance is measured at identical angle of detection (AOD) with that of incidence (AOI) while diffuse reflectance is measured at the other AOD. While dense WO_3_ thin film shows much higher specular reflectance (when AOD-AOI ≈ 0°), diffuse reflectance is dominant with almost no specular reflectance peak for the WO_3_ 3D NH arrays as shown in Fig. 6f. This indicates that 3D NH arrays strongly scatter incident light as compared with the dense film, which is consistent with the simulation results. Therefore, 3D NH arrays are suitable for light harvesting layers providing high probability for light absorption.

### Applications of 3D NH Arrays

#### Passive Optical Components

**Fig. 7:**
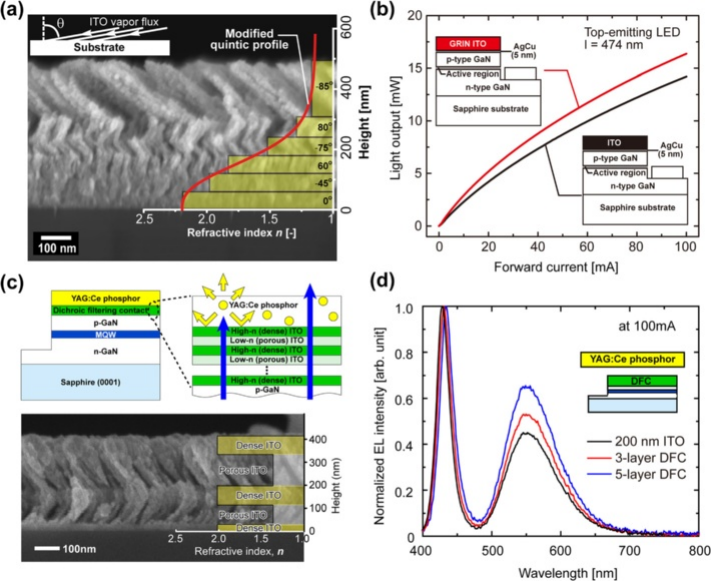
Passive optical components. **a** Cross-sectional SEM image of GRIN ITO AR coating with modified-quintic refractive index profile on a GaInN LED. The GRIN ITO AR coating consists of six ITO layers with pre-determined refractive indices for optimum AR characteristics [65]. **b** Average light-output power of 30 representative top-emitting GaInN LED chips with dense ITO coating and with GRIN ITO AR contact [65]. **c** Schematic illustration of a phosphor-converted dichroic white LED with a DFC and the structure of the DFC consisting of an alternating stack of high-refractive-index (dense) ITO and low-refractive-index (porous) ITO. Cross-sectional SEM image shows five-layer DFCs made of a single material, ITO. The measured refractive indices of dense (*n* = 2.08) and porous (*n* = 1.35) ITO layers by ellipsometry are shown as a function of height from the substrate [66]. **d** Measured spectra of white LEDs with 200-nm-thick ITO contact, three-layer, and five-layer DFCs using the same phosphor layer for all three configurations [66]

The reflection of incident light at the interface of two optical media, for example, semiconductor and air, is one of the major optical loss mechanisms in various optoelectronic devices including solar cells and light-emitting diodes (LEDs) [67, 68]. Such optical loss by reflection can be significantly reduced by introducing an antireflection (AR) layer whose refractive index gradually decreases from the refractive index of the active semiconductor layer to that of the surrounding medium, typically air. OAD has been proposed to fabricate a graded-refractive-index (GRIN) AR coating layer. As an example of this concept, a six-layer GRIN AR coating made of a single material, ITO, has been applied to GaInN LEDs [65] to increase the light extraction by reducing the reflection at the interface between GaN and air. Figure 7a shows a cross-sectional SEM of the GRIN ITO AR contact consisting of six ITO layers having tailored refractive indices; the refractive index of the bottom layer is 2.19, which closely matches that of GaN while that of the top layer is 1.17, matching that of the air. Therefore, the thin film structure matches the refractive index of air and substrate and is expected to have the excellent AR characteristics. Figure 7b shows the average light-output power of 30 representative top-emitting GaInN LEDs with conventional ITO transparent contact, and with GRIN ITO AR contact, as a function of forward current. At an injection current of 20 mA, the LEDs with GRIN ITO AR coating show 24.3 % higher light output than the reference LEDs with dense ITO contact. Besides, near-perfect AR reflection coatings with broadband and omni-directional characteristics enabled by GRIN configuration using OAD and their applications to various solar cells have been demonstrated [61, 63, 64, 69].

In addition to the multi-layered AR coatings with graded refractive indices, thin films consisting of alternating layers of low- and high-refractive indices fabricated by OAD can be used for high-performance optical filters and distributed Bragg reflectors [62] made of a *single* material. White LEDs based on the combination of a GaInN-based blue LED and a yellow phosphor layer suffer from a low phosphor conversion efficiency (PCE) because a significant amount of yellow fluorescence from the yellow phosphor layer is emitted toward the blue LED chip where the fluorescence is partially absorbed. Incorporated conductive dichroic-filtering contacts (DFCs) made of a single ITO by OAD was reported to multifunction as a blue-transmitting but yellow-reflecting optical filter as well as a low-resistance ohmic contact to GaN, thus improving the PCE of the white LEDs [66]. Figure 7c shows a schematic description of a phosphor-converted white LED with a conductive DFC which has alternating stacks of low-refractive-index nanoporous ITO and high-refractive-index dense ITO, and its cross-sectional SEM image. DFC layers are composed of five-layer ITO thin film structures having clear interfaces. Figure 7d represents the emission spectra of white LEDs with three different contacts normalized with respect to the blue electroluminescence peak so that the enhancement of yellow fluorescence can be clearly seen. Compared to the LED with reference ITO contact, yellow fluorescence of LEDs with DFCs is improved due to the enhanced transmission of blue electroluminescence through the DFC and the increased reflection of the downward yellow fluorescence.

#### Photoelectrodes for Solar-Energy-Harvesting Cells

**Fig. 8:**
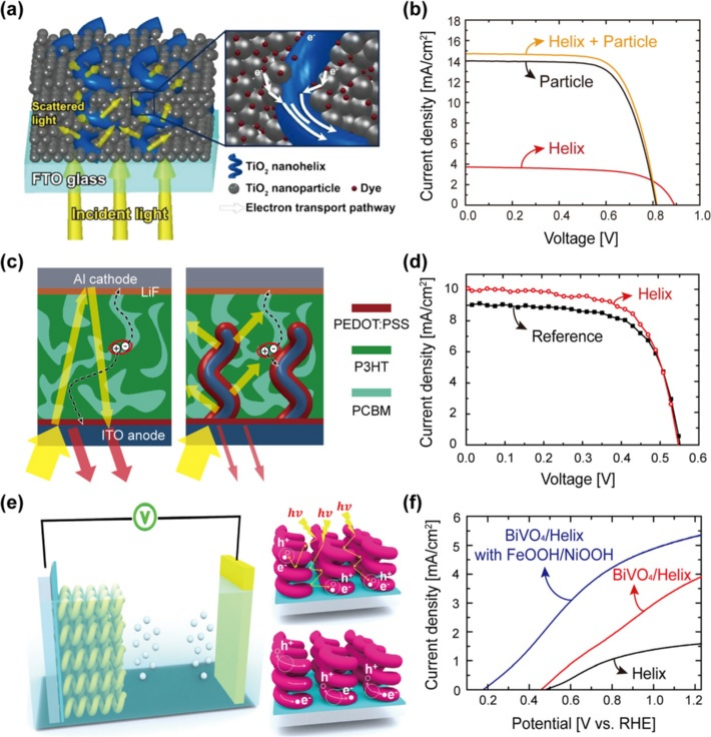
Photoelectrode applications. **a** Schematic description of light scattering and electron transport in a NH-nanoparticle (NP) photoanode. The magnified view shows an enhanced transport of electrons generated from dye molecules, through the infiltrated TiO_2_ NPs followed by a TiO_2_ NH [21]. **b** *J*-*V* curves for the DSSCs with various photoanodes [21]. **c** Schematic drawings of bulk heterojunction (BHJ) solar cells with a typical planar photoanode and an ITO NH photoanode [23]. **d** *J*-*V* curves of BHJ solar cells with typical planar photoanode and with ITO NH photoanode [23]. **e** Schematic drawings of PEC cells with WO_3_ NH electrode [24]. **f** *J*-*V* curves of PEC cells with photoanodes of helical WO_3_, WO_3_/(W, Mo)-BiVO_4_, and WO_3_/(W, Mo)-BiVO_4_/FeOOH/NiOOH [24]

Performances of solar-energy-harvesting systems such as solar cells and photoelectrochemical cells are closely related to the nanostructure of photoelectrodes since the photoelectrodes play two important roles in solar-energy-harvesting processes: (i) absorption of solar radiation to create photo-generated carriers which determines “light harvesting efficiency,” and (ii) transport of photo-generated carriers into the external circuit determining “charge collection efficiency.” The geometrical shape and size of a photoelectrode mainly affect the light harvesting efficiency, while the charge collection efficiency is strongly influenced by the micro-structural properties of the photoelectrode, i.e., whether the micro-structure can provide photo-generated carriers with “express ways” for efficient transport with little recombination loss. Unfortunately, there is a trade-off, in general, between the light harvesting and the charge collection efficiencies. Therefore, a rational design of the nanostructures for photoelectrodes has been an important issue to improve both the efficiencies simultaneously, alleviating or even breaking the trade-off. Arrays of 3D NHs fabricated by OAD technique have been proposed as a suitable photoelectrode due to their enhanced light scattering properties (Fig. 6) and efficient transport of photo-generated carriers with reduced recombination losses at grain boundaries enabled by excellent crystallinity (Fig. 4), thus improving both optical and electrical properties simultaneously. Larger light scattering cross section in 3D NH arrays results in longer traveling distances of incident light within the active region and thus enhances the light harvesting efficiency. In addition, 3D NHs can provide direct pathways for photo-generated carriers through highly crystalline NHs with much less grain boundaries compared to sintered nanoparticle electrodes, leading to much improved charge collection efficiency. Improvements of energy conversion efficiencies in dye (or quantum dot)-sensitized solar cells ((Q)DSSCs) [21, 22], organic photovoltaic cells (OPVs) [3], and photoelectrochemical cells (PECs) [24] by applying arrays of metal-oxide NHs as photoelectrodes have been demonstrated. Figure 8 shows schematic diagrams of three devices—a DSSC, an OPV, and a PEC with photoelectrodes based on arrays of metal-oxide NHs, and their performances. In DSSCs (Fig. 8a,b), the array of TiO_2_ NHs infiltrated with TiO_2_ nanoparticles as a multifunctional photoelectrode shows 6.2 % enhancement of the power conversion efficiency in comparison with the DSSC with the conventional nanoparticle photoelectrode due to simultaneously improved light scattering and carrier transport properties by TiO_2_ NH arrays, while maintaining a comparable surface area accessible for dye molecules by the infiltrated TiO_2_ nanoparticles [22]. In the case of the OPV, ITO NH arrays used as a multifunctional photoelectrode (Fig. 8c) result in strong light harvesting, which is attributed to effective antireflection coating as well as light scattering effects, and enhanced carrier transport through highly crystalline ITO NHs. The optical and electrical improvements enabled by the ITO NH in the OPV increase its power conversion efficiency by 10 % as shown in Fig. 8d [23]. The combination of effective light scattering, improved charge transport properties, and an enlarged surface area accomplished by the BiVO_4_-decorated WO_3_ NH photoelectrode in the photoelectrochemical solar water splitting system (Fig. 8e) leads to a very high photocurrent density (~5.35 ± 0.15 mA cm^−2^) at 1.23 V versus the reversible hydrogen electrode as shown in Fig. 8f [24].

#### Sensing Layers for Various Sensors

3D nanostructured thin films fabricated by OAD exhibit high porosities and high surface-to-volume ratio, providing a great potential to be used for active layers of chemical sensors due to their giant surface areas for chemical reactions as well as easiness of in-and-out of chemical species being detected [15, 16, 70–73]. Hawkeye et al. demonstrated a photonic-crystal optical humidity sensor using OAD [70]. Layers with alternating refractive indices having high and low density were fabricated for active layers whose spectral features shift when relative humidity changes. Beckers et al. fabricated SiO_2_ nanostructured films by OAD for active layers of selective alcohol sensors, successfully distinguishing methanol, ethanol, and 2-propanol [71]. In addition to chemical sensors, an array of Cr zigzag nanosprings for a pressure sensor was fabricated by Kesapragada et al. [73]. The Cr zigzags exhibit a reversible change in resistivity upon loading and unloading, due to a compression of the nanosprings which causes them to physically touch their neighbors, indicating their potential as pressure sensors.

**Fig. 9:**
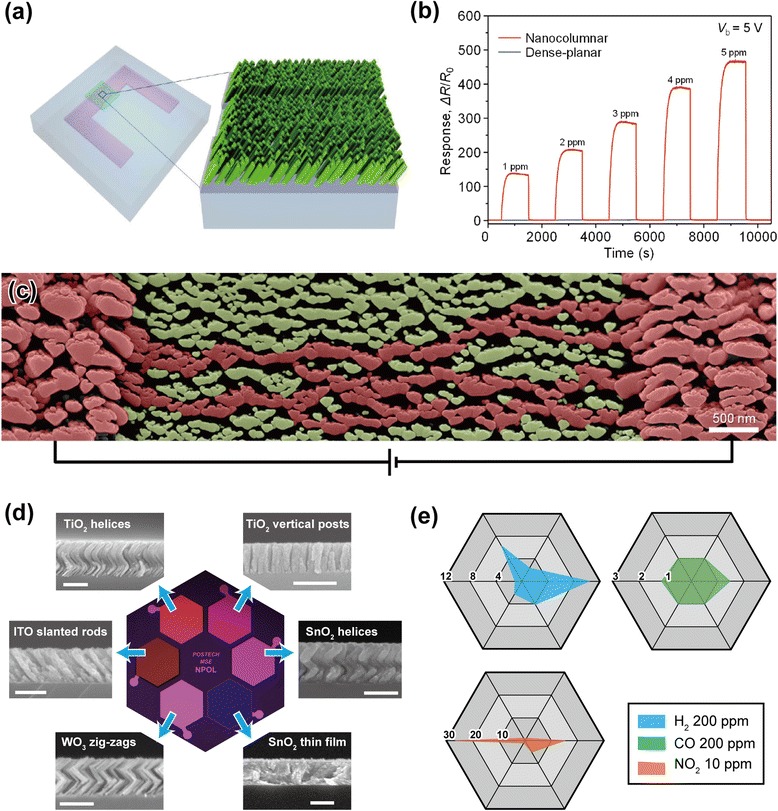
Chemical sensor applications. **a** Schematic showing fabricated transparent sensors based on nanocolumnar oxide films [16]. **b** Sensing transients of the dense-planar and nanocolumnar WO_3_ thin film sensors to 1–5 ppm NO_2_ at an applied bias voltage of 5 V [16]. **c** 40°-tilted SEM image of nanocolumnar WO_3_ film between and on ITO IDEs. Parts highlighted in *reddish color* indicate localized current pathways which meander with narrow necks [16]. **d** Optical microscopy (OM) image of the fabricated e-nose chip after the deposition of six different layers and corresponding cross-sectional SEM images (*scale bar* = 300 nm) [15]. **e** Radar chart patterns of the prototype e-nose chip for H_2_ 200 ppm, CO 200 ppm, and NO_2_ 10 ppm. Each *vertex* of the radar corresponds to each sensor of the array, where the devices were measured at 150 °C with 0.2 V applied bias [15]

In addition, all oxide transparent chemoresistive sensors have been realized, with ultra-high sensitivity, extremely low power consumption, and excellent long-term stability utilized by uniquely nanostructured metal-oxide thin films by OAD [16]. A fabricated sensor is schematically shown in Fig. 9a, consisting of ITO interdigitated electrodes (IDEs) with nanocolumnar WO_3_ films. The linear and ultra-high response of the nanocolumnar sensor to 1–5 ppm of NO_2_ is observed, while the response of the dense-planar sensor is negligibly small and sluggish, as shown in Fig. 9b. This is because of self-activation effects, utilizing much more active chemical reactions between sensing layers and target gases. The nanocolumnar film temperature was found to rise to 139 °C at 5 V bias voltage by self-activation, which serves as a very efficient self-activated microheater with minimal power consumption. Figure 9c shows a 40°-tilted SEM image of nanocolumnar WO_3_ film deposited between and on the ITO IDEs, showing unique geometry with disconnected current pathways or percolating pathways meandering through narrow necks (20- to 40-nm width). For this geometry, electron flow is constricted, leading to increased joule heating. 3D NH arrays as sensing layers have been also suggested for chemical sensors [15]. TiO_2_ NH arrays have been found to show much enhanced sensing performances compared to the reference (TiO_2_ thin film). This is enabled by giant surface areas of TiO_2_ NH films and Debye-length (~22 nm for TiO_2_)-comparable diameter of each NH. In addition to a single gas sensor device, the prototype electronic-nose (e-nose) chip consisting of six gas sensors with different nanostructures (thin film, NHs, vertical posts, and zigzags) or sensing materials (TiO_2_, ITO, SnO_2_, and WO_3_) has been demonstrated as shown in Fig. 9d, demonstrating the excellent compatibility with current microelectronics processes, thus a great potential to be used for multifunctional integrated circuit chips. Radar charts of gas sensitivity toward H_2_, CO, and NO_2_ gas species measured from the prototype e-nose are shown in Fig. 9e.They indicate that each gas sensor has its specific sensitivity pattern for different gas molecules, offering the function of discriminating various gas species. The proposed technology, we believe, allows a tremendous freedom in design and realization of multi-sensor chips for a ubiquitous-sensor-network-based future society.

## Conclusions

This review article introduced recent studies on 3D nanostructured thin films fabricated by OAD technique, which is suitable for producing a wide range of 3D nanostructures with excellent controllability, reproducibility, and compatibility with current microelectronics processes. By tilting the substrate during OAD, the initial growth fluctuation (nuclei) forms a self-shadowed region against highly directional vapor flux, creating a self-organized nanoporous thin film. The geometrical shape of the 3D nanostructures can be tailored by adjusting the combination of three parameters (*r*, *θ*, *ϕ*) during the deposition. The 3D nanostructures fabricated by OAD show unique structural and optical properties including excellent crystallinity after proper annealing, accurate tunability of refractive indices down to near 1.0, and strong light scattering effects due to their large scattering cross section. These unique structural and optical features can be utilized to enhance performances of various systems when they are applied to passive optical components, photoelectrodes in solar-energy-harvesting cells, sensing layers in diverse sensors and their integrated forms—electronics noses, and other future devices.
